# Evolutionary pathway and genetic mechanisms of fin-to-limb transition in vertebrates

**DOI:** 10.3389/fcell.2026.1784849

**Published:** 2026-04-14

**Authors:** Andrey V. Bayramov, Dmitry N. Mednikov, Andrey G. Zaraisky

**Affiliations:** 1 Shemyakin-Ovchinnikov Institute of Bioorganic Chemistry, Russian Academy of Sciences, Moscow, Russia; 2 Koltzov Institute of Developmental Biology of Russian Academy of Sciences, Moscow, Russia; 3 Borissiak Paleontological Institute, Russian Academy of Sciences, Moscow, Russia

**Keywords:** AER, autopod, digit arch theory, fin-to-limb transition, Gegenbaur, *Hox* genes, limb bud, metapterygium

## Abstract

The transformation of paired appendage structure from aquatic fins to terrestrial limbs represents a pivotal event in vertebrate evolution, underpinning the colonization of land by early tetrapods. This transition involved profound morphological and genetic modifications, particularly in the distal limb region known as the autopod and in the dorsoventral plane of paired appendages. Recent advances in paleontology, comparative and functional genomics, as well as evo-devo studies have shed light on several key events and evolutionary pathways and have improved our understanding of the direction of changes in regulatory mechanisms underlying the fin-to-limb transition. In this review, we aim to summarize current knowledge on limb evolution, with particular emphasis on studies of phylogenetically pivotal vertebrate groups - cartilaginous fishes and chondrosteans, which represent basally diverging evolutionary lineages of extant vertebrates, as well as sarcopterygians, the group of lobe-finned fishes most closely related to tetrapods. We consider the principal hypotheses concerning the prerequisites for vertebrate terrestrialization, key aspects in the search for structural homology between the morphological elements of fins and limbs, as well as the genetic mechanisms of spatial limb bud development described to date and the possible modifications of these mechanisms associated with the transformation of ancestral fins into pentadactyl terrestrial limbs.

## 1 Introduction

The limbs of terrestrial vertebrates are homologous to the paired fins of fish, as both are evolutionarily derived from the locomotory appendages of ancestral vertebrates and share multiple developmental mechanisms (Yano and Tamura, 2013). During ontogeny, the limb bud arises as a lateral outgrowth of the body wall and subsequently differentiates into a three-dimensional structure under the influence of signaling centers and regulatory factors (Sheeba et al., 2016a). Proximodistal patterning of the limb bud is directed by signals from the apical ectodermal ridge (AER), instructed by Fgf8 (Kengaku et al., 1998; Fernandez-Teran and Ros, 2008; Lu et al., 2008). The anteroposterior axis is specified by the zone of polarizing activity (ZPA), which secretes Sonic hedgehog (Shh) (Harfe et al., 2004; Zeller et al., 2009), while dorsoventral patterning is regulated by *Lmx1b*, *Wnt7a*, and *En1* (Loomis et al., 1998; Castilla-Ibeas et al., 2024). Despite these fundamental similarities, fins and limbs differ markedly in morphology, complicating the identification of true homologies among all elements of these appendage types (Yano and Tamura, 2013).

**FIGURE 1:**
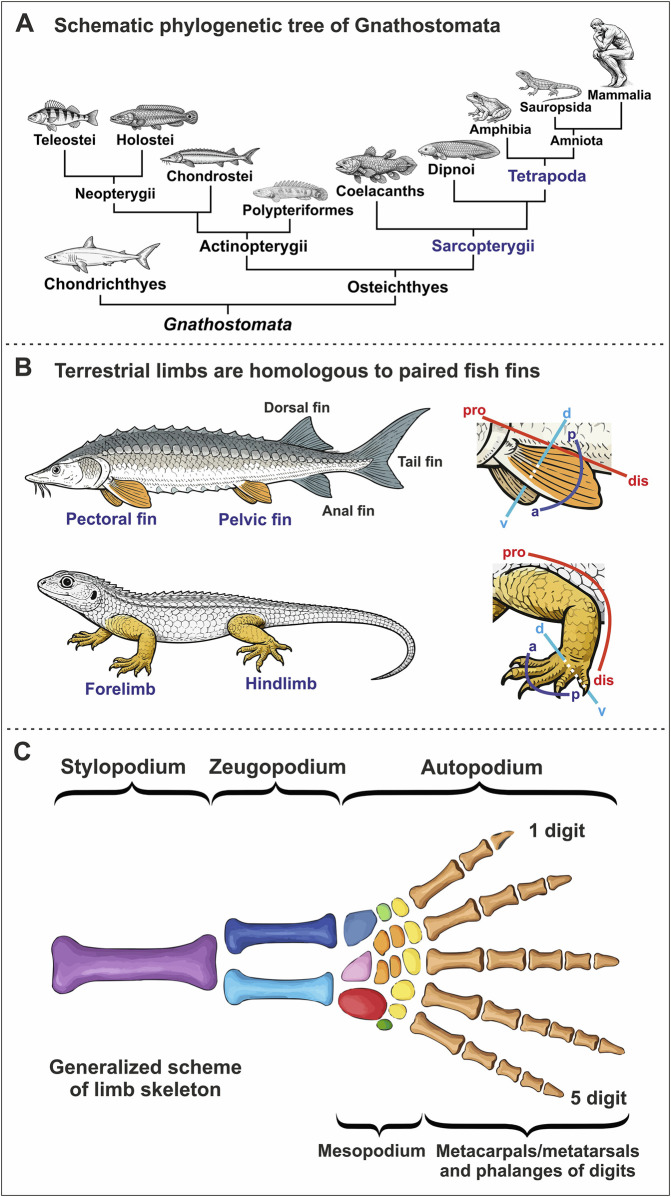
Limbs and Phylogeny of Gnathostomata. **(A)** A schematic phylogenetic tree of the major gnathostome taxa. The fin-to-limb transition occurred within the Sarcopterygii during the transition to Tetrapoda. **(B)** The basic body plan of fish includes unpaired (dorsal, anal, and caudal) and paired (pectoral and pelvic) fins. The limbs of terrestrial vertebrates are homologous to paired fins. These paired appendages are three-dimensional structures characterized by proximo-distal, antero-posterior, and dorso-ventral axes. a - anterior, d - dorsal, dis - distal, p - posterior, pro - proximal, v - ventral. **(C)** The scheme of the generalized pentadactyl tetrapod limb. The proximo-distal structure of the limb includes the stylopodium, zeugopodium, and autopodium, which is subdivided into the mesopodium, metacarpals/metatarsals, and the phalanges of the digits (digital rays). Purple is humerus/femur; dark blue is radius/tibia; blue is ulna/fibula; light blue is radiale/tibiale; pink is intermedium; red is ulnare/fibulare; orange is centralia; yellow is carpalia/tarsalia distalia; light green is praepollex/praehallux; green is postminimus; brown is metacarpals/metatarsals and phalanges of digits.

Understanding the evolutionary origin of tetrapod limbs requires integrating developmental mechanisms with comparative morphology across key phylogenetic groups. Among extant vertebrates, the pectoral fins of cartilaginous fishes (Chondrichthyes), as phylogenetically the most basally divergent group of jawed vertebrates, are expected to retain ancestral paired appendages features and developmental mechanisms (Cole and Currie, 2007). The skeletal organization of cartilaginous fish fins includes a full set of endoskeletal elements (basalia and radials) and dermal ceratotrichia (Riley et al., 2017). The basal section comprises three elements - the propterygium, mesopterygium, and metapterygium - a configuration that may correspond to the fundamental structure of the ancestral fin (Bayramov et al., 2024; Thompson et al., 2021). Within the evolutionary lineage of Actinopterygii, there is a trend toward the reduction of the posterior basal element, the metapterygium, which is fully realized in teleosts (Coates, 1994; Thompson et al., 2021). Paleontological evidence suggests that tetrapod limbs evolved from the paired fins of lobe-finned fishes through transformation of the endoskeleton and reduction of dermal elements (Schneider and Shubin, 2013; Stewart et al., 2020; Cloutier et al., 2020). In extant sarcopterygians, as in tetrapods, only a single basal element - the metapterygium - remains at the base of the appendage (Coates and Cohn, 1998). Thus, actinopterygian fins and tetrapod limbs represent divergent evolutionary modifications of a common ancestral appendage. This divergence complicates direct comparisons between actinopterygian fins and tetrapod limbs and highlights the importance of studying phylogenetically pivotal taxa, highlighting the importance of developmental studies in key phylogenetic groups, including cartilaginous fishes (basally divergent gnathostomes), sturgeons (basally divergent actinopterygians), and lungfishes (closest extant relatives of tetrapods) (Figure 1A). The rapid expansion of genomic and transcriptomic data in these groups has recently facilitated a more detailed reconstruction of appendage evolution in vertebrates (Amemiya et al., 2013; Marra et al., 2019; Du et al., 2020; Meyer et al., 2021; Wang et al., 2021; Cheng et al., 2021; Bi et al., 2021; Yamaguchi et al., 2023; Marlétaz et al., 2023; Wagner et al., 2023; Sendell-Price et al., 2023; Seixas et al., 2023; Schartl et al., 2024).

A number of detailed and comprehensive studies have been devoted to synthesizing data on the formation of terrestrial limbs, including several key review articles (Shubin et al., 1997; Clack, 2009a; Cass et al., 2021; Varga and Varga, 2022). In this review, we aimed to integrate and expand upon the existing framework by incorporating new paleontological and genetic evidence, with a particular focus on representatives of such groups as Chondrichthyes, Chondrostei, and Dipnoi in the context of the transformation of ancestral fins into terrestrial limbs. An important question that is repeatedly raised in studies investigating the developmental mechanisms underlying specific structural features of terrestrial limbs - such as proximodistal organization, dorsoventral asymmetry, synovial joints, and the distal region including the mesopodium and autopodium - is whether these “innovations” arose directly during the formation of terrestrial-type limbs or whether the prerequisites for their development were already present at the genetic and morphological levels prior to the transition of vertebrates to land. A number of studies published in recent years, which we attempt to consider in this review, substantially deepen our understanding of these issues.

## 2 Paired and unpaired fins in gnathostomes

Before considering the fossil record and developmental mechanisms underlying the fin-to-limb transition, it is useful to briefly outline the basic anatomical organization of vertebrate fins. The basic body plan of gnathostome fishes includes paired (pectoral and pelvic) and unpaired, or median (dorsal, anal, and caudal), fins (Figure 1B; Coates, 2003; Zhu et al., 2012; Letelier et al., 2018; Sansom et al., 2013; Charest et al., 2023; Bayramov et al., 2024). Since, according to currently available paleontological evidence, unpaired fins appeared earlier in vertebrate evolution than paired ones, they may be considered potential evolutionary precursors of paired appendages (Freitas et al., 2006; Freitas et al., 2014). In representatives of Teleostei, duplication of the unpaired anal and caudal fins has been demonstrated following disruption of the expression of one of the paralogs of the *Chordin* gene (Abe et al., 2014).

At the same time, unpaired and paired fins are not fully identical in their structural organization. While the proximodistal and anteroposterior axes are present in both fin types, the dorsoventral asymmetry characteristic of the pectoral and pelvic fins - located laterally on the body - has no direct counterpart in the structure of the dorsal, anal, and caudal fins, which are symmetrical relative to the median sagittal plane of the body (Zdral et al., 2026; Dale et al., 2026). During the transition of vertebrates to land, paired fins were transformed into terrestrial limbs capable of supporting the body weight of the animal during locomotion on a solid substrate. The paired appendages became more robust, and their flexibility was ensured by joints connecting the segments of the proximodistal axis of the limb (Mansuit and Herrel, 2021; Clack, 2012).

The question of fin-to-limb transition has been investigated since the 19th century, based on evidence from comparative morphology and paleontology (Shubin and Alberch, 1986; Clack, 2009b).

Morphologically, the skeletal structure of tetrapod limbs comprises three proximodistally arranged segments: (1) the stylopod, a proximal segment with a single bone (the humerus in forelimbs or femur in hindlimbs) articulating with the limb girdle; (2) the zeugopod, an intermediate segment consisting of two parallel bones (radius and ulna in the forelimb, tibia and fibula in the hindlimb); and (3) the autopod, a distal segment encompassing the mesopodium (wrist or ankle) and digits (Figure 1C). While the number of endoskeletal elements in the stylopod and zeugopod (1 and 2, respectively) remains evolutionarily conserved, the autopod is morphologically more variable and accounts for much of the structural diversity observed in vertebrate limbs. This variation has enabled the evolution of specialized forms such as wings and flippers, allowing for diverse modes of locomotion beyond terrestrial walking (Sheeba et al., 2016a; Langellotto et al., 2018).

During the fin-to-limb transition, several major morphological changes appear to have been fundamental. These include the reduction and eventual loss of dermal fin rays and the elaboration of the distal endoskeleton leading to the formation of digits composed of endochondral bones (Nakamura et al., 2016; Stewart et al., 2020; Esteve-Altava et al., 2019). These transformations are most pronounced in the distal elements, where the flexible fin blade is replaced by a stiff, jointed autopod (Tulenko et al., 2017; Tanaka, 2016). In addition, dorsoventral asymmetry of limb structure became established - or, according to recent data, was substantially enhanced - likely in association with the functional demands of supporting and transmitting body weight during locomotion on a solid substrate (Castilla-Ibeas et al., 2024; Stewart et al., 2020). These morphological modifications were accompanied by transformations at the genetic and regulatory levels, which we consider in the sections below.

## 3 Functional and morphological prerequisites and environmental hypotheses of terrestrialization

The transition from aquatic to terrestrial environments, which occurred during the Devonian period, represents a dramatic ecological shift that required profound modifications across multiple organ systems (Schmalhausen, 1968; Shubin et al., 2009; Clack, 2012; Jung et al., 2018). Here, however, we focus specifically on changes in the morphology and function of paired appendages and the molecular mechanisms that may have driven these transformations. Throughout, the term “terrestrial limb” refers to an appendage adapted for movement on solid substrates, as opposed to fins adapted for locomotion in water.

Paleontological evidence suggests that limbs with distinctively tetrapod-like features, including a digit-bearing autopod, may have evolved in aquatic organisms prior to the colonization of land, supporting the “limb before terrestriality” hypothesis (Coates and Clack, 1990; Coates and Clack, 1995; Coates, 1996; King et al., 2011; Dickson et al., 2021). Current reconstructions of morphological changes during the water-to-land transition are informed by a series of well-preserved fossils, including *Tiktaalik*, *Elpistostege*, *Acanthostega*, *Ichthyostega*, *Pederpes* and some others (Shubin et al., 2006; Coates, 1996; Pierce et al., 2012; Clack and Finney, 2005; Cloutier et al., 2020; Dickson et al., 2021). The restructuring of fin endoskeletons and musculature to support substrate-based locomotion is thought to have set the stage for terrestriality in Devonian tetrapod ancestors (Pierce et al., 2012; 2013; Ahlberg, 2019).

Several hypotheses have been proposed to explain the fin-to-limb transition in lobe-finned ancestors. Barrell (1916) and Sushkin (1922) posited that arid Devonian climates led to the desiccation of water bodies, forcing ancestral tetrapods onto land and promoting the evolution of limbs. In contrast, Watson (1926) argued for a primarily aquatic origin of tetrapods, citing features of Carboniferous amphibians (embolomeres) - short, weak limbs, massive elongated bodies, loose pelvic attachments, and well-developed lateral line canals - as evidence of obligate aquatic life. Later finds such as *Acanthostega* and *Ichthyostega* further supported this view, retaining caudal fin structures such as supraneurals and lepidotrichia (Clack, 2011; 2012), and even internal gills in the case of *Acanthostega* (Coates and Clack, 1991; Clack, 2012). On the other hand, studies over the past few decades have shown that finned tetrapodomorphs such as *Tiktaalik* and *Elpistostege* already possessed a number of morphological features that had previously been considered typically tetrapod (Shubin et al., 2006; Shubin et al., 2014; Cloutier et al., 2020). For example, *Tiktaalik* exhibited not only an enlarged endochondral pectoral girdle and a reduced dermal series of ossifications (cleithrum, clavicle, anocleithrum, and supracleithrum), as well as synovial joints in its pectoral fins (Shubin et al., 2006), but also a very large pelvic girdle - much larger than in other tetrapodomorph fishes - and pelvic fins comparable in size to the pectoral ones (Shubin et al., 2014). The pronounced enlargement of the pelvic girdle in this fish suggests that the trend toward an increased propulsive role of the hind appendages had already emerged in finned tetrapodomorphs (Shubin et al., 2014). These findings not only blur the clear boundary between fishes and tetrapods but also indicate that the principal set of tetrapod characteristics was already established in fully aquatic animals.

Romer (1958) proposed that limbs evolved to facilitate short-distance overland travel between drying water bodies during climatic fluctuations - a “desert hypothesis” - but emphasized that true terrestrial adaptation occurred later in the more favorable environments of the Late Carboniferous. Goin and Goin (1956), drawing on amphibian biology, countered that extant amphibians do not migrate during droughts, but rather estivate in moist substrate, similar to lungfish. Orton (1954) advanced a burrowing hypothesis, interpreting tetrapod feet as adaptations for digging. Niedźwiedzki et al. (2010) described fossil trackways dated to 395 million years ago, suggesting limb function in intertidal habitats - an “intertidal hypothesis” - though this interpretation remains contentious (e.g., Kuznetsov, 2021).

Reevaluation of Devonian red sandstones in the mid-20th century suggested these sediments formed under humid rather than arid conditions, more akin to modern tropical forests (Tatarinov et al., 1965). These insights informed Inger’s (1957) “humid habitat” hypothesis, proposing that limbs evolved in saturated forest-floor environments similar to modern Amazonian and Southeast Asian floodplains, where many amphibious fishes exhibit terrestrial behaviors. According to Inger, terrestriality was driven by high aquatic population densities and the availability of unoccupied semi-aquatic niches (Inger, 1957; Tatarinov et al., 1965). A related “woodland hypothesis” is supported by Retallack (2011), who identified transitional fossils in deposits corresponding to wet floodplain forests. In this view, limbs evolved as locomotory adaptations for navigating shallow, vegetated pools and inundated terrain.

Notably, throughout fish evolution, the ability to move across solid substrates has independently arisen multiple times among representatives of various taxonomic groups, including both cartilaginous and bony fishes (Lucifora and Vassallo, 2002; Porter et al., 2022; Dickson and Pierce, 2018; Renous et al., 2011). In addition to the evolution of terrestrial limbs as structures optimized for locomotion on solid ground, a variety of alternative solutions have also been explored. Movement can be achieved via undulatory body axis motions, as observed in the reedfish *Erpetoichthys calabaricus* (Polypteridae), or by using paired fins, as in mudskippers (*Periophthalmus* spp., Gobiiformes) or frogfishes (Lophiiformes). Polypterids employ their pectoral fins for locomotion (Foster et al., 2018), whereas lungfishes and the ambulatory cavefish *Cryptotora thamicola* use pelvic fins (King et al., 2011; Aiello et al., 2014; Flammang et al., 2016). *Channa* (Anabantiformes), one of the largest extant fishes capable of terrestrial excursions and comparable in body size to Devonian tetrapods such as *Acanthostega* and *Tulerpeton*, moves across solid substrates using its spiny anal fin (Kuznetsov, 2021). Some species, such as the walking catfish (*Clarias* spp., Siluriformes), may combine axial and fin-based movements during locomotion on land (Pace and Gibb, 2014).

## 4 In search of structural homology and transitional forms from fins to limbs

While ecological hypotheses attempt to explain the environmental context of terrestrialization, the structural transformation of fins into limbs is most clearly documented in the fossil record and comparative anatomy. The identification of structural homology between the paired fins of fishes and the limbs of terrestrial vertebrates, as well as the search for transitional forms between these types of vertebrate appendages, represent classical problems in evolutionary biology with a long and rich intellectual history (Long and Gordon, 2004; Cloutier et al., 2020; Molnar et al., 2021).

**FIGURE 2:**
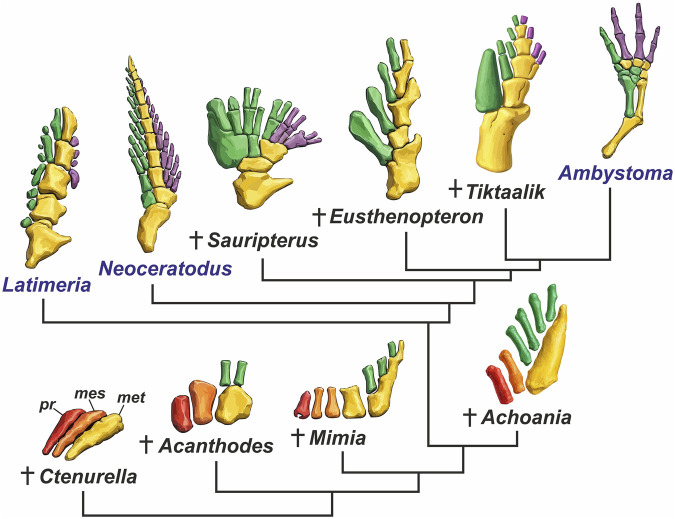
Skeletons of the pectoral appendages of various groups of gnathostomes: placoderm fish *Ctenurella* (from Jarvik, 1964, with modifications), acanthodian fish *Acanthodes* (from Coates, 1994, with modifications), primitive actinopterygian fish *Mimia* (from Gardiner, 1984, with modifications), stem sarcopterygian fish *Achoania* (from Zhu and Yu, 2009, with modifications), extant actinistian fish *Latimeria* (from Millot and Anthony, 1958, with modifications), extant lungfish *Neoceratodus* (from Rosen et al., 1981, with modifications), tetrapodomorph fish *Sauripterus* (from Davis et al., 2004, with modifications), tetrapodomorph fish *Eusthenopteron* (from Andrews and Westoll, 1970a, with modifications), tetrapodomorph fish *Tiktaalik* (from Shubin et al., 2006, with modifications), extant tetrapod *Ambystoma* (from Romer and Parsons, 1986, with modifications concerning the preaxial nature of the thumb according to Woltering and Meyer, 2015). Abbreviations: propterygium is highlighted in red, mesopterygium is highlighted in orange, metapterygium or metapterygial axis are highlighted in yellow, preaxial radials of metapterygium are highlighted in green, postaxial radials of metapterygium are highlighted in purple.

In paleontological studies, the first well-preserved pectoral fins discovered were those of *Sauripterus* and *Eusthenopteron* (Figure 2). The pectoral fin of *Eusthenopteron* was first studied by Whiteaves in 1883. It contains a clearly defined segmented axis composed of four endoskeletal mesomeres, decreasing in size distally. From the first three mesomeres, single preaxial radials branch off, while the distal fourth mesomere supports two small radials (Andrews and Westoll, 1970a). If radials are interpreted as digit precursors, then the total number (five) corresponds exactly to the number of digits in tetrapod limbs. However, unlike digits, these radials are short, monolithic, and unsegmented.

In 1843, J. Hall described the pectoral girdle and fin skeleton of *Sauripterus*, noting its striking similarity to the tetrapod limb (Hall, 1843). The fin of *Sauripterus* is more complex than that of *Eusthenopteron*: although its axis consists of only three mesomeres, it contains more radials that bifurcate dichotomously, each supporting two terminal radials (Andrews and Westoll, 1970b). As with *Eusthenopteron*, the first mesomere connecting the fin to the girdle is homologized with the tetrapod humerus.

The homology of two elements articulating with the first mesomere in *Sauripterus* and other sarcopterygians with the ulna and radius has been contentious (Shubin and Alberch, 1986). A.N. Sewertzoff considered the large first preaxial radial of *Sauripterus* to be a second element of the metapterygial axis and homologized it with the ulna, interpreting the second mesomere as the most proximal radial and homologous with the radius (Sewertzoff, 1926). Most researchers, however, have regarded the first radial as the homologue of the radius and the second mesomere as the ulna (Broom, 1913, among others). A key argument for homologizing the ulna with the second mesomere is Westoll’s “ulnar pyramid” model (Shubin and Alberch, 1986). In tetrapod limb development, postaxial dominance is evident: the postaxial branch, beginning at the ulna or fibula, gives rise to most mesopodial elements and all digits. The preaxial branch includes the radius/tibia and radiale/tibiale, and possibly praepollex/praehallux elements (Shubin and Alberch, 1986). This pattern of postaxial dominance is also characteristic of Paleozoic sarcopterygian fins, supporting homologies between the second fin segment and the zeugopodial bones of tetrapods (Friedman et al., 2007). Only in fossil porolepiforms and extant lungfishes is the zeugopod of the pectoral fin represented by a single element. In living lungfishes, this single element results from fusion of the humeral and ulnar homologues during development (Druzinin, 1933; Rosen et al., 1981; Jude et al., 2014).

Homologizing the distal elements of sarcopterygian fins with tetrapod autopodial bones has proven far more challenging. Most researchers homologize the third mesomere of the metapterygial axis with the tetrapod ulnare/fibulare (Shubin, 1995), regarding the presence of ulnare/fibulare as a synapomorphy of all sarcopterygians (Rosen et al., 1981; Chang, 1991; Schultze, 1991). The second preaxial element branching from the second mesomere is homologized with the intermedium (Shubin, 1995). Some researchers argue that the intermedium is an apomorphy restricted to osteolepiforms, elpistostegalians, and tetrapods, and absent in other sarcopterygians, including fossil and extant lungfishes (Schultze, 1991; Jude et al., 2014). However, in coelacanths - basal to both dipnomorphs and tetrapodomorphs - a second preaxial radial is present, suggesting that the intermedium was likely ancestral to all sarcopterygians, with a secondary loss in dipnomorphs (Chang, 1991; Friedman et al., 2007; Jude et al., 2014). Thus, the presence of homologues for the humerus, ulna, radius, ulnare, and intermedium likely constitutes a shared trait across Sarcopterygii (Rosen et al., 1981).

The search for homologs of autopodial elements more distal than the intermedium and ulnare in tetrapods among the distal skeletal elements of lobe-finned fish fins has historically presented significant challenges. Gregory and Raven (1941), and shortly thereafter Westoll (1943), examining the pectoral fin of *Eusthenopteron* as a starting point for the formation of the terrestrial limb, proposed that the digits and distal elements represent novelties that arose along the postaxial margin of the metapterygial axis, which was bent anteriorly. Westoll introduced the terms “archepodium” for the complex of ancestral mesopodial elements present in fish ancestors, and “neopodium” for the new elements of the wrist, ankle, and digits that emerged in tetrapods (Westoll, 1943).

Paleontological discoveries of recent decades have substantially expanded our knowledge of the limb structure of the earliest tetrapods and the fins of various Paleozoic sarcopterygians, particularly the elpistostegalians, which are considered the sister group to tetrapods. The biserial (as detailed below), asymmetric pectoral fins composed of numerous skeletal elements in *Panderichthys*, *Tiktaalik*, and *Elpistostege* filled the morphological gap separating the typical fish fin from the terrestrial limb (Boisvert et al., 2008; Shubin et al., 2006; Cloutier et al., 2020). Many structural features previously regarded as characteristic exclusively of tetrapods - such as postaxial digits and elements of the mesopodium - were shown to be present in some fishes as well.

Analyses of the transformation of dermal fin rays in fossil tetrapodomorphs along the series *Sauripterus taylori* - *Eusthenopteron foordi* - *Tiktaalik roseae* revealed clear trends toward consolidation and shortening of the fin rays, as well as the development of dorsoventral asymmetry in the dermal skeleton. In *Tiktaalik*, the rays become shortened, cease to branch and segment, and the fin web is strongly reduced; consequently, the rays no longer extend far beyond the endoskeleton. In this taxon, dorsoventral asymmetry becomes particularly pronounced: dorsal rays cover a substantial portion of the distal elements of the endoskeleton, whereas ventral rays terminate much more proximally (Stewart et al., 2020). In addition, all species in this series show differences in the cross-sectional area of dorsal and ventral rays, with dorsal rays in *Tiktaalik* being two to three times thicker than the ventral ones. These changes in the dermal skeleton correspond to the adaptation of fins to loading associated with substrate support and indicate the transformation of the dorsoventral axis of the appendage as an important evolutionary direction during the fin-to-limb transition.

## 5 Metapterygial axis and the digital arch theory

Karl Gegenbaur was the first to develop a theory for the evolutionary origin of the tetrapod limb from an ancestral fin. In 1865, through the study of the endoskeleton of pectoral fins in a wide range of fishes, Gegenbaur identified a common key element he termed the ***metapterygium*** (Gegenbaur, 1865; Grandel, 2003). The metapterygium is a large endoskeletal element situated along the posterior margin of the fin in many primitive fish groups and typically supports additional endoskeletal structures - the radials. Gegenbaur observed that in many cartilaginous fishes the metapterygium is segmented. He considered the sequentially arranged segments of the metapterygium (mesomeres), forming the ***metapterygial axis***, as the main axis of the fin. He proposed that the most primitive type of fin consisted of a single metapterygium with radials articulated on one side, which he termed the *archipterygium* (Gegenbaur, 1870; Gegenbaur, 1872). Archipterygia with a single row of radials were described as uniserial, whereas those with two rows were termed biserial.

Gegenbaur’s concept of the metapterygial axis became a key reference point for comparisons between fin and limb endoskeletons. However, both Gegenbaur himself and his numerous followers, in their search for the metapterygial axis in the tetrapod limb, encountered difficulties in identifying it within the autopodium and proposed various interpretations of its course in the limbs of tetrapods.

**FIGURE 3:**
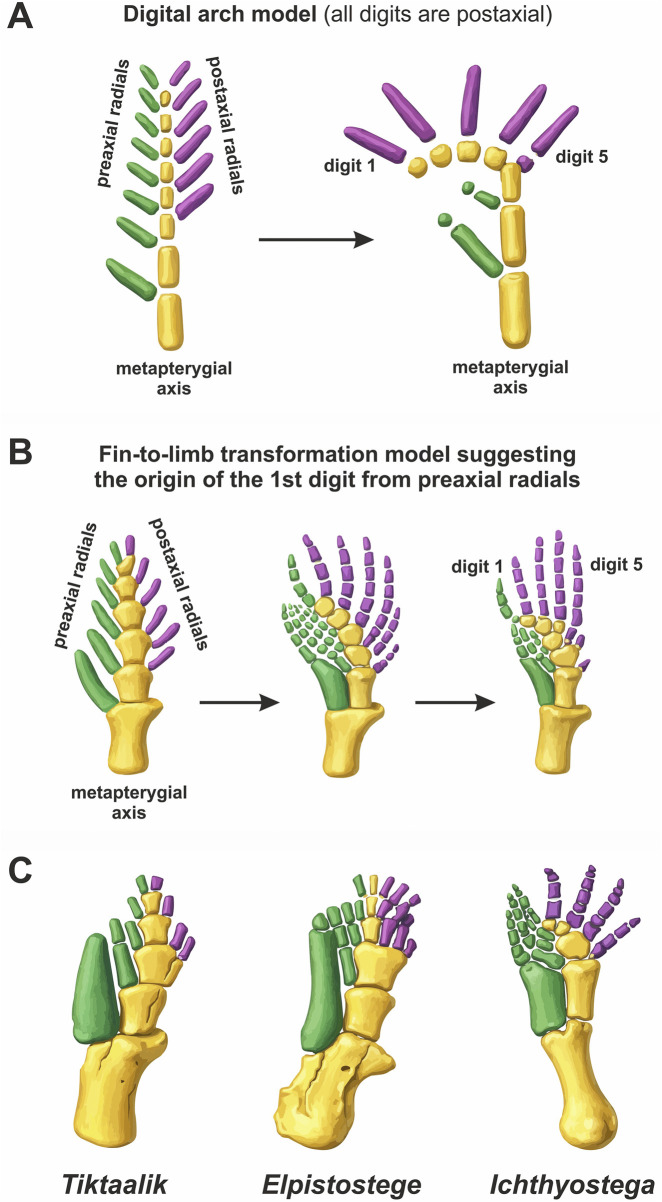
Shubin-Alberch digital arch theory: schemes and fossil forms. **(A)** scheme of the metapterygial axis transformation into a digital arch, where all digits are postaxial elements of the limb (according to Shubin and Alberch, 1986, with modifications). **(B)** the variant of the transformation in which the first digit is a derivative of the preaxial radial, and all other digits are derivatives of the postaxial radials (according to Mednikov, 2018, with modifications). **(C)** The pectoral fin of elpistostegalia *Tiktaalik* (according to Shubin et al., 2006, with changes). The pectoral fin of the elpistostegalia *Elpistostege* (according to Cloutier et al., 2020, with changes). The hindlimb of the Devonian tetrapod *Ichthyostega* (Coates and Clack, 1990, with modifications). The elements of the metapterygial axis are highlighted in yellow, the preaxial elements of the limb skeleton are highlighted in green, and the postaxial elements are highlighted in purple.

In 1986, Shubin and Alberch, after extensive study of limb development across diverse tetrapod groups, proposed that the metapterygial axis continuation in the limb corresponds to a row of distal mesopodial elements bearing digits. This typically curved arc-shaped row of distal elements, representing a distinct developmental pattern of linked limb elements, was termed the “digital arch” by Shubin and Alberch, following Schmalhausen (1910) (Shubin and Alberch, 1986). According to their hypothesis, the metapterygial axis does not pass through a digit nor between digits - as previously thought by many - but transversely through the mesopodium. The ancestral tetrapod fin axis bent anteriorly, positioning its distal end at the anterior edge of the limb. The proximodistal sequence of mesomer formation in the fin axis became a posterior-to-anterior sequence of distal element formation. Digits are homologs of postaxial radials of the biserial fin, while the radius/tibia, intermedium, and centralia correspond to preaxial radial elements (Figure 3A; Shubin and Alberch, 1986). According to the digital arch theory, the ancestral tetrapod fin was biserial and asymmetric. During fin-to-limb transition, as the metapterygial axis curved anteriorly, distal preaxial rays were reduced (Figures 3A,B; Mednikov, 2009; Mednikov, 2014; Mednikov, 2018).

Recent paleontological discoveries also support the digital arch theory’s inference that the fin, ancestral for the limb, was biserial, asymmetric, and highly segmented. The pectoral fins of the closest tetrapod relatives - the elpistostegalians - were biserial, with a comparatively long axis consisting of at least five mesomeres in *Tiktaalik* and *Elpistostege* (Figure 3C; Shubin et al., 2006; Cloutier et al., 2020). In *Elpistostege*, some radials, both preaxial and postaxial, were segmented like true digits, and the first preaxial ray bore a distinct element homologous to the radiale, further aligning the fin with the terrestrial limb (Cloutier et al., 2020). The *Panderichthys* pectoral fin, reassessed via tomography, was also found to be more complex than previously thought and exhibited an asymmetric biserial organization (Boisvert et al., 2008). Devonian biserial fins with moderately long axes were also present in *Shoshonia* (a coelacanth) and *Penetlandia* (a lungfish) (Friedman et al., 2007; Jude et al., 2014). The *Shoshonia* fin was notably asymmetric and included more mesomeres (at least six) than the coelacanth (*Latimeria*) fin (four) (Friedman et al., 2007). This moderately long, asymmetric biserial fin may represent the ancestral condition for lobe-finned fishes in general (Friedman et al., 2007), and the immediate tetrapod ancestors retained this ancient pattern. A recent description of the pectoral fin of another elpistostegalian, *Qikiqtania* (Stewart et al., 2022), showed that some closest tetrapod relatives underwent fin axis shortening to three mesomeres and likely secondary loss of postaxial radials in response to adaptation to open-water habitats.

Despite the archetypal similarity to tetrapod limbs, elpistostegalian pectoral fins retained typical fish characteristics. In *Tiktaalik*, these included lepidotrichia, unsegmented postaxial radials, and the absence of a typical tetrapod autopodium subdivided into morphologically and functionally distinct mesopodial and digital phalangeal elements. In *Elpistostege*, some postaxial radials were segmented like true digits, increasing resemblance to tetrapods; however, the fin axis was only weakly preaxially curved, and three of its five postaxial radials remained unsegmented (Cloutier et al., 2020). The *Elpistostege* fin also lacked clear morphological differentiation into short mesopodial elements and elongated digital phalanges. Its distal complex of small bones can be compared to an extensive flexible mesopodium, with a fan of lepidotrichia replacing digits.

The inherited biserial arrangement of the limb, with two digit groups - preaxial and postaxial - may have persisted in some tetrapods (Mednikov, 2009; 2014; Woltering and Meyer, 2015). The Devonian amphibian *Ichthyostega* retained a clearly biserial limb organization comparable to elpistostegalian fins, consistent with the digital arch concept (Mednikov, 2014; 2018) (Figure 3C). The three preaxial digits of *Ichthyostega* were noticeably thinner than the four postaxial digits and tightly appressed as in a fin. Similar to the preaxial radials in the fins of many sarcopterygians, the preaxial digits of the *Ichthyostega* hindlimb show a progressive reduction in size from the first to the third (Mednikov, 2014). Another Devonian tetrapod, *Acanthostega gunnari*, discovered in the same deposits as *Ichthyostega*, although considered the most completely known among Devonian tetrapods (Coates, 1996), is notably inferior to *Ichthyostega* in the degree of autopodial preservation. In both the forelimb and, particularly, the hindlimb of *Acanthostega*, many mesopodial elements are missing from known specimens, apparently being cartilaginous in life (Coates and Clack, 1990; Coates, 1996). Because of this, as well as due to postmortem displacement of elements (including the phalanges), a reliable complete reconstruction of the autopodium is impossible for either the forelimb or the hindlimb of *Acanthostega*. Nevertheless, both the fore- and hindlimbs of *Acanthostega* bear two preaxial digits that differ markedly in morphology from the remaining digits, giving the limbs of this animal a distinctly biserial appearance (Mednikov, 2014). Some preaxial digits may have persisted in modern tetrapods (Woltering and Meyer, 2015). Many anurans and some mammals exhibit a well-developed pre-first digit (praepollex and/or praehallux) that may comprise two or more phalanges and even bear a keratinous claw, as in *Xenopus* (Hayashi et al., 2015) and the hoofed lemming (Manzii, 1950).

Another preaxial digit, arising from a preaxial radial, may be the first digit (thumb) (Woltering and Meyer, 2015). This digit differs from the other four by several features and shows properties characteristic of preaxial limb elements such as the radius or tibia. In urodeles, the first digit develops independently from digits 2–5, ontogenetically linked to the curved anterior end of the digital arch, and may represent the homolog of the last preaxial radial of the ancestral fin, arising from the last mesomere of the metapterygial axis (Mednikov, 2009; 2018). Thus, modern tetrapod limbs, possessing both preaxial (pre-first and first digit) and postaxial (two to five) digits, have retained the biserial pattern characteristic of *Elpistostege* and *Ichthyostega*.

It has been shown that during limb development in urodeles, a gradual preaxial bending of the digital arch occurs. At early mesenchymal stages, the arch is only slightly curved and resembles the axis of a fin, whereas in later larval stages, it acquires an increasingly pronounced preaxial inclination. Digits 2–5 develop on the postaxial side of the weakly curved digital arch, arranged one above the other, resembling in their arrangement the postaxial radials of a biserial fin. As the arch bends, the digits assume the typical limb configuration, forming a transverse row relative to the long axis of the limb (Mednikov, 2009).

In summary, data on the development of modern tetrapod limbs (especially urodeles), alongside the morphological features of the limbs of the earliest tetrapods and the fins of elpistostegalians, are highly consistent with the Shubin-Alberch theory of the digital arch as a homolog of the distal part of the ancient metapterygial axis of the ancestral terrestrial limb fin (Mednikov, 2009; 2014).

At the same time, the digital arch theory has been challenged by several experimental studies indicating both the early specification of the limb bud (prior to cartilage condensation) and the developmental independence of distal limb elements, including the digits, from proximal ones (Wolpert and Hornbruch, 1990; Cohn et al., 2002; Dudley et al., 2002; Hamrick, 2007). The hypothetical mechanism of digital arch formation proposed by Shubin and Alberch (1986), which suggested that the arch arises through sequential branching and segmentation of prechondral elements, has also been questioned. In reality, branching of prechondral elements is not observed during limb development, calling the Shubin - Alberch model of digital arch formation into question (Cohn et al., 2002).

However, the refutation of this particular hypothetical mechanism (branching of the rudiments) does not negate the very possibility of the existence of a digital arch and its homologation with the distal part of the metapterygial axis of the ancestral fin. In the modern lungfish *Neoceratodus*, during the development of its paired fins, there is also no dichotomous branching of the rudiments during the formation of mesomers and radials (Holmgren, 1933; Joss and Longhurst, 2001; Johanson et al., 2007), however, this does not prevent the formation of a typical axial fin with a biserial structure. The digital arch itself has been repeatedly described by researchers in various tetrapods (Schmalhausen, 1910; Schmalhausen, 1915; Schmalhausen, 1964; Shubin and Alberch, 1986; Mednikov, 2009). Although Schmalhausen, the discoverer of the digital arch, noted that the transverse connections between the distal elements of the mesopodium are a “specific acquisition of terrestrial vertebrates” and arose in connection with access to land to ensure the “supporting function of the palm and foot” he also emphasized that these connections between the distals arise very early in the development of the limb (Schmalhausen, 1964). In representatives of primitive Caudata, the formation of the digital arch occurs really very early, at the mesenchymal stage, long before the appearance of prechondral or cartilaginous precursors of skeletal elements, which may indicate the phylogenetic antiquity of the digital arch and its continuity with the metapterygial axis of the sarcopterygian fins (Mednikov, 2009). Finally, the digital arch hypothesis is supported by its great predictive value, especially for paleontology (Mednikov, 2014). The prediction that the fins ancestral to the tetrapod limb should have a complex biserial structure (Shubin and Alberch, 1986) was fully justified with the discovery of skeletal remains of fins of such forms as *Tiktaalik* and, especially, *Elpistostege* (Shubin et al., 2006; Cloutier et al., 2020).

## 6 Anatomical features and differences in the mechanisms of development of paired fins and limbs

**FIGURE 4:**
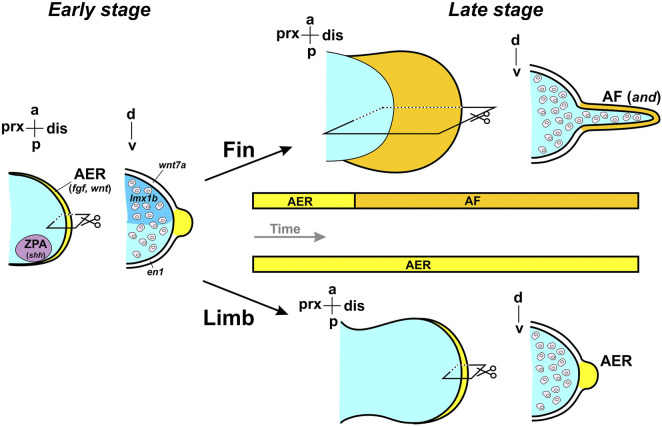
Features of fin and limb bud formation. The divergence in the developmental mechanisms of fins and limbs becomes more pronounced at later stages. Exoskeletal elements of the fin originate from the apical fold (AF).

In both structure and developmental mechanisms, tetrapod limbs and paired fish fins share significant similarities, underscoring their homology. At the same time, they exhibit some notable differences at the genetic and cellular levels (Figure 4). The growth and differentiation of limb and fin buds are regulated from early stages by the activity of two signaling centers: the apical ectodermal ridge (AER) and the zone of polarizing activity (ZPA), which are sources of Fgf and Shh signals, respectively (Delgado and Torres, 2016). Differences in the ontogeny of fins and limbs become more evident at later stages, particularly in the formation of the distal elements (Leite-Castro et al., 2016).

The AER, a thickening of the ectoderm at the distal tip of the limb bud, remains active during the proximodistal outgrowth of the appendage. While an AER also forms during fin development (Norton et al., 2005), it is subsequently transformed into an apical fold (AF), which participates in the patterning of the distal fin region (Yano and Tamura, 2013). Structurally, the distal region of the fin differs from that of the tetrapod limb in being composed of exoskeletal elements - dermal fin rays - that form the fin blade. These dermal skeletal elements, like the proximal skeletal components, are derived from the lateral plate mesoderm (LPM) (Onimaru et al., 2011; Enny et al., 2020; Tzung et al., 2023). The endoskeletal components of fins, namely the basal and radial elements, develop via endochondral ossification: mesenchymal condensations differentiate into cartilage, which is later replaced by mineralized bone. In contrast, dermal skeletal elements mineralize directly without a cartilaginous intermediate. Paired fins thus integrate both dermal and endochondral ossification, whereas tetrapod limbs contain only endochondral skeletal elements. In teleosts pectoral fins and girdle, are formed via perichondral ossification during early embryonic development (Hall, 2007; Wei et al., 2025).

A key feature of distal fin development is the transition of the apical ectodermal ridge into the apical fold (AER-to-AF transition) (Thorogood, 1991). As the AF develops, mesenchymal cells from the LPM migrate into it and contribute to the formation of the fin rays (Grandel and Schulte-Merker, 1998; Enny et al., 2020). Throughout the evolutionary transition from fins to limbs, an expansion of endoskeletal elements is accompanied by a reduction of dermal fin rays, reflecting a general shift toward an increased contribution of endochondral endoskeletal structures (Coates, 1994; Stewart et al., 2020). This trend is evident in the fossil record, but similar developmental trajectories are observed in extant taxa, such as lungfishes - the closest living relatives of tetrapods (see below) (Yano and Tamura, 2013). It is proposed that this structural shift may be driven by heterochronic changes, specifically a delay in the timing of the AER-to-AF transition during fin development. In *Danio* (a representative of evolutionarily derived teleosts), experimental delay of the AER-to-AF transition results in increased endoskeletal contribution to the fin (Yano et al., 2012; Yano and Tamura, 2013). Mechanistically, the processes governing the AER-to-AF transition are thought to inhibit endochondral ossification, and a reduction in these processes may have facilitated the evolutionary transformation from fin to limb.

Despite these differences, the AER of developing limbs and the AER + AF complex of fin buds exhibit a high degree of genetic similarity, including the expression of marker genes such as *wnt2b*, *dlx2*, *dlx5a*, *sp8*, and *sp9* (Neumann et al., 1999; Ng et al., 2002; Kawakami et al., 2004). Fgf signaling, emanating from the AER, plays a crucial role in the outgrowth and patterning of both fins and limbs (Yano and Tamura, 2013). Such similarity in genetic mechanisms may reflect the homology of fins and limbs, highlighting the possibility that they arose through the evolutionary transformation of a single ancestral appendage.

## 7 Dipnoi as transitional state model

Among extant vertebrates, lungfishes represent a particularly informative model for studying the evolutionary transition from fins to limbs. Following the discovery of the Australian lungfish *Neoceratodus*, whose paired fins exhibit a biserial structure, Gegenbaur (1872) regarded this fin type as the most primitive. Huxley (1876), considering lungfishes the group most closely related to tetrapods, attempted to derive the tetrapod limb from a *Neoceratodus*-like fin. Given their phylogenetic position, lungfishes, and especially *Neoceratodus*, have recently become one of key objects of genetic investigation into the mechanisms underlying the fin-to-limb transition in tetrapods and continue to attract considerable attention from researchers investigating various aspects of tetrapod limb evolution, as they represent the closest living sarcopterygians to tetrapods (Figure 1A; Joss and Longhurst, 2001; Diogo et al., 2016; Woltering et al., 2020; Cloutier and Ahlberg, 1996; Broughton et al., 2013; Liang et al., 2013). The phylogenetic position and morphological structure of paired fins makes the study of lungfish fins particularly interesting as potential transitional forms in the evolution of terrestrial limbs (Amaral and Schneider, 2018; Hirasawa et al., 2021).

Only a few relict species of lobe-finned fishes survive today: two species of coelacanths and six species of lungfishes (Clement, 2019). The genomes of representatives of these groups have been sequenced in recent years (Amemiya et al., 2013; Meyer et al., 2021; Wang et al., 2021; Schartl et al., 2024). Genome synteny analyses confirm that lungfishes are the closest living relatives of terrestrial vertebrates (Meyer et al., 2021). Three genera of extant lungfishes persist: the South American *Lepidosiren*, the African *Protopterus*, and the Australian *Neoceratodus*.

Morphologically, *Neoceratodus* resembles fossil forms such as *Ceratodus*. The fins of *Neoceratodus* exhibit the structural plan typical of sarcopterygians, characterized by a central metapterygial axis, at the base of which homologs of the humerus, radius, and ulna can be distinguished (Woltering et al., 2020). A fin organized according to the biserial archipterygium pattern contains segmented and unbranched radials that resemble the limb architecture of ancestral terrestrial vertebrates. Tetrapod digits are likewise segmented and unbranched structures (Coates, 1994).

The fins of *Neoceratodus* also exhibit key limb-specific organizing centers and regulatory circuits: the zone of polarizing activity (ZPA), governed by the Shh - Gli3 regulatory loop, and the apical ectodermal ridge (AER), regulated by Fgf signaling (Woltering et al., 2020). The regulatory mechanisms underlying limb development will be discussed in more detail in the following sections.

Studies of the fin musculature in modern lobe-finned fishes - lungfish and coelacanth - have suggested that the characteristic tetrapod skeletomuscular proximal limb phenotype, particularly the presence of superficial and deep muscle layers, dorsal and ventral muscle groups, and pre- and postaxial muscle masses, was already present in the last common ancestor (LCA) of extant sarcopterygians in the Silurian (Diogo et al., 2016). The difference in the muscular organization of the fins of Sarcopterygii compared to tetrapod limbs is that in fishes, muscle bundles attach primarily to the bones, whereas in tetrapods, tendons are associated with the joints, which allows for more precise movements. In cartilaginous fishes and the bichir (one of representative of basal Actinopterygii; Figure 1A), the dorso-ventral organization is represented by two muscle masses - dorsal and ventral (Wilhelm et al., 2015; Diogo and Ziermann, 2015). In contrast, in *Latimeria* and *Neoceratodus*, the dorsal and ventral muscles are significantly more complex and represent mirror images of each other, demonstrating an example of derived similarity (Diogo et al., 2016; Miyake et al., 2016). According to the hypothesis of Diogo et al., the sarcopterygian LCA probably possessed at least two layers of adductor and abductor muscles that were partially segmented proximo-distally at the level of each joint. The authors propose that the LCA of extant sarcopterygians likely already exhibited the basic tetrapod limb muscle phenotype in both the pectoral and pelvic appendages, with the exception of the characteristic tetrapod autopod, which arose later in evolution during the development of the terrestrial limb.

## 8 Formation of the three-dimensional limb bud structure

Compared with fins, limbs generally constitute more massive three-dimensional structures with a robust endoskeleton, capable of supporting and moving the weight of the animal in space (Yano and Tamura, 2013). Following the primary specification of the prospective limb field along the anterior-posterior axis of the body, an epithelial-to-mesenchymal transition (EMT) and intense proliferation of cells within the somatopleure of the lateral plate mesoderm (LPM) lead to the expansion of a mesenchymal cell mass enveloped by ectoderm. This results in the outward bulging of the forming limb bud from the body surface (Gros and Tabin, 2014). Subsequent development of limb rudiments with a fully developed three-dimensional structure involves differentiation along the three orthogonal axes: proximodistal, anteroposterior and dorsoventral axes (McQueen and Towers, 2020; Castilla-Ibeas et al., 2024; Cooper et al., 2011; Zeller et al., 2009; Sheeba et al., 2016b; Delgado and Torres, 2016; 2017). Below we review current data on the principal signaling factors involved in the development of the limb bud, including those studied in representatives of phylogenetically pivotal vertebrate groups, as well as modifications of regulatory modules that may have occurred during the evolutionary transformation of ancestral fins into terrestrial limbs.

### 8.1 Proximodistal axis

Proximodistal growth and differentiation are primarily driven by **Fgf signals** secreted from the distally located apical ectodermal ridge (AER), which forms under the influence of *fgf8* expression in the ectoderm (Ohuchi et al., 1997). In both fin and limb buds, the AER expresses a conserved suite of *Fgf* genes, including *fgf8* and *fgf4* (Grandel et al., 2000; Neumann et al., 1999; Nomura et al., 2006; Boulet et al., 2004; Lu et al., 2006; Lin and Zhang, 2020). In the pectoral fins of *Danio*, the epithelial-mesenchymal signaling factor *fgf10* induces *fgf16*, which is required for cell proliferation and differentiation in the mesenchyme and in the AER of the fin buds (Itoh and Ohta, 2014). *Fgf16*, in turn, induces the expression of *fgf4* and *fgf8* in the AER (Nomura et al., 2006).

In the limbs of terrestrial vertebrates, the activity and functional roles of *Fgf4*, *Fgf8*, *Fgf9*, and *Fgf17* have been described (Lu et al., 2006; Mariani et al., 2008). Blocking *Fgf* signaling disrupts normal limb development (Lewandowski et al., 2000; Moon and Capecchi, 2000; Nomura et al., 2006). The importance of the AER for limb development and proximo-distal specification was demonstrated in experiments involving the removal of the AER at different stages of limb development in chick embryos (Saunders, 1948). Further evidence comes from natural AER knockout - like phenotypes observed in snake hindlimbs. In pythons, forelimbs fail to be induced due to the anterior expansion of *hoxc8* and *hoxb5* expression domains in the paraxial mesoderm and lateral plate mesoderm (LPM). Hindlimbs are induced but fail to form a functional AER; in the absence of AER-derived signals, they develop only as rudimentary structures (Cohn and Tickle, 1999).

The formation of the proximal limb element, the stylopod, is characterized by expression of ***Meis* genes**, presumably activated by retinoic acid (RA) diffusing from surrounding cells (Mercader et al., 2000). Growth and development of zeugopodial cells depend on *hoxa11* activity, modulated by Fgf signaling from the AER (Capdevila et al., 1999; Mercader et al., 1999; Davis et al., 1995). Expression domains of *meis* and *hoxa11* in limbs are spatially segregated between the stylopod and zeugopod, serving as markers of proximodistal differentiation (Yadav et al., 2023; Delgado et al., 2020; Langellotto et al., 2018).

In chondrichthyan fins *meis* and *hoxa11* expression patterns are also distributed proximally and distally, indicating the likely presence of basic mechanisms for proximodistal limb bud patterning early in jawed vertebrate evolution (Sakamoto et al., 2009). However, a discrete physical boundary between proximal and distal domains, as seen in tetrapod limbs, is absent in chondrichthyan fins. In sturgeon and teleost fins, *meis* and *hoxa11* expression domains are not segregated along the proximodistal axis and their active expression phases occur early, prior to endoskeletal element formation (Tulenko et al., 2017; Langellotto et al., 2018).

**FIGURE 5:**
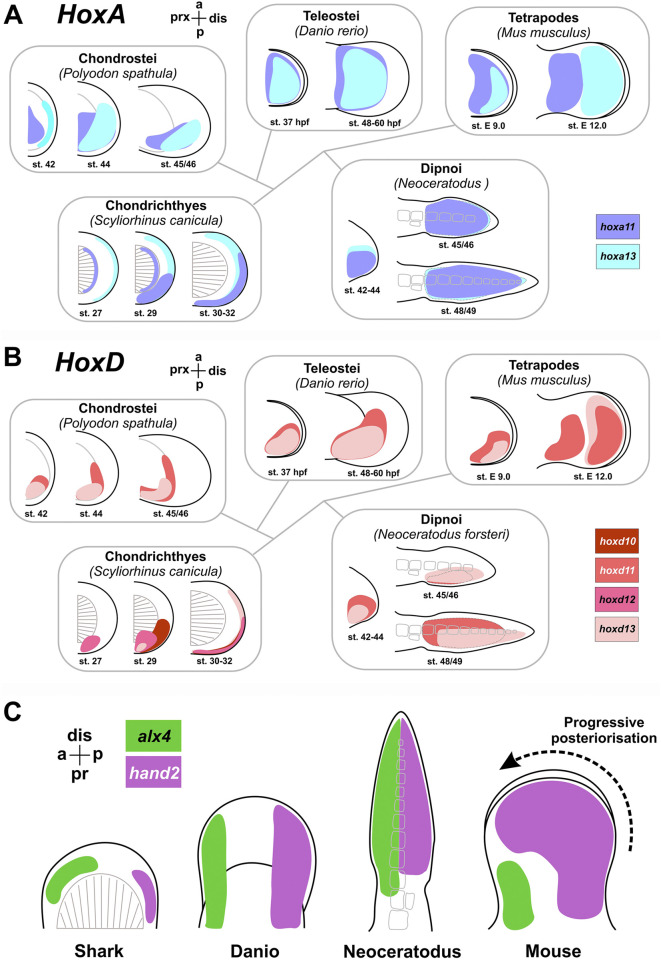
Regulatory mechanisms of proximo-distal and anterio-posterior specification of limb bud. **(A,B)** expression of *Hoxa* and *Hoxd* cluster genes across different vertebrate evolutionary groups (adapted from Tanaka et al., 2021; Tulenko et al., 2016; Tulenko et al., 2017; Metscher et al., 2005; Langellotto et al., 2018; Nakamura et al., 2016; Sakamoto et al., 2009; Woltering et al., 2020). **(C)** Progressive posteriorization (after Onimaru et al., 2015; Woltering et al., 2020).

During fin chondrogenesis in *Neoceratodus*, *meis1* and *meis3* are diffusely expressed in the proximal third of the fin, with lower levels distally along the fin axis (Langellotto et al., 2018). A proximodistal gradient of *meis* expression is evident, peaking proximally where the stylopodium is formed. *Hoxa11* in *Neoceratodus* is expressed distally in the fin bud and is absent proximally. Thus, the spatial distribution of *meis* and *hoxa11* expression in *Neoceratodus* pectoral fins resembles that of tetrapod limbs. However, a notable feature in *Neoceratodus* fins is the overlapping expression domains of *hoxa11* and *hoxa13* (Figure 5A; Langellotto et al., 2018).

In contrast, tetrapod limbs exhibit non-overlapping, segregated expression of *hoxa11* and *hoxa13* during late development stages, coinciding with the autopod formation (Figure 5A). Late *hoxa11* expression localizes proximally in the zeugopod-forming domain, while *hoxa13* is active more distally in the presumptive autopod. Teleost fins lack such segregation; *hoxa11* and *hoxa13* expression domains overlap (Sordino et al., 1995). Similarly, sturgeon (*Polyodon*) fins also do not display separation of *hoxa11* and *hoxa13* expression zones (Davis et al., 2007).

Genes of ***Hoxd* cluster** also represent key regulators of limb bud patterning. Similar to their role in embryonic body axis differentiation, the expression patterns of *Hox* genes in limbs exhibit temporal and spatial collinearity (Tarchini and Duboule, 2006). Prior to limb bud formation, *hoxd9* is expressed in the LPM within the presumptive limb induction region (Cohn et al., 1997). As the limb bud develops, alongside persistent *hoxd9* expression, neighboring cluster genes (*hoxd10 - hoxd13*) are sequentially activated, displaying nested expression domains with *hoxd13* marking the most posterior domain (Tarchini et al., 2006). This pattern is characteristic of early stages of both fin and limb bud formation and is shared by fore- and hindlimbs (Figure 5B).

In studies of terrestrial limb development, it was found that at later stages of differentiation, a second phase of activation of *Hoxd* gene expression is observed, which was not detected in the development of the teleosts fins (Zakany and Duboule, 1999). Spatially, this late (second) phase is restricted to the distal limb bud, does not overlap with the proximal expression domains of the early phase, and temporally coincides with differentiation of the autopod skeletal elements (mesopod and digit primordia). Notably, *Hoxd* expression during this late phase displays inverse collinearity compared to the early phase, such that the anterior boundary of *hoxd13* expression extends more anteriorly relative to other *Hoxd* genes (opposite to the early phase) (Shubin et al., 2009). The late phase of *Hoxd* expression is regulated independently of the early phase via distinct enhancers (Spitz et al., 2003).

The emergence of biphasic *Hoxd* expression during evolution is proposed as a potential regulatory innovation that may have triggered autopod formation. In *Chondrichthyes*, as representatives of the basally divergent lineage of jawed vertebrates, biphasic *Hoxd* expression has also been observed (Figure 5B; Freitas et al., 2007). Similarly to tetrapod limbs, the late phase in shark fins is localized distally and exhibits inverse collinearity. This suggests the presence of regulatory prerequisites for distal limb development already early in jawed vertebrate evolution and implies a likely secondary loss of these regulatory modules in teleost fishes.

A comparable *Hoxd* expression pattern was initially described in another archaic jawed vertebrate group - the sturgeons (exemplified by *Polyodon*) (Davis et al., 2007); however, these findings were not confirmed by subsequent studies (Tulenko et al., 2016). In *Polyodon*, late-stage *Hoxd* expression in fin fold mesenchyme overlaps with expression of *and1*, a gene regulating exoskeletal element development (Tulenko et al., 2016). Thus, the *Hoxd* expression pattern in *Polyodon* aligns with that seen in teleosts and may represent a synapomorphy of ray-finned fishes (Ahn and Ho, 2008).

Experimental overexpression of one paralog of the *hoxd13* gene (*hoxd13a*) in *Danio* resulted in distal expansion of proliferating chondrogenic tissue expressing marker genes *sox9a* and *col2a1*, accompanied by a reduction of the fin fold with decreased expression of *and1* and *fgf8a* (Freitas et al., 2012). These morphological changes resemble evolutionary transformations observed during the fin-to-limb transition (Sordino et al., 1995; Freitas et al., 2012).

In the lungfish *Neoceratodus*, only the late phase of *hoxd13* expression was detected, occurring after the formation of axial endoskeletal elements - the stylopod and zeugopod (Johanson et al., 2007). *Hoxd13* expression was observed in the post-axial domain preceding the onset of radials formation, suggesting a potential role of *hoxd13* in radial development in *Neoceratodus*, analogous to its involvement in digit formation in terrestrial vertebrates (Tarchini and Duboule, 2006; Johanson et al., 2007). These findings provide support for the homology of lobe-finned fish radials and the autopod elements of the tetrapod limb.

In *Danio* (teleosts), a mutant lines named *rephaim* (*reph*) and *wan* exhibits additional endoskeletal elements termed “intermediate radials” developing in the posterior region of the pectoral fin (Hawkins et al., 2021). These elements lie between posterior proximal and distal radials and do not articulate with the girdle, morphologically resembling zeugopod elements. Their development is regulated by altered activity of *waslb* and *vav2* genes expressed in early fin mesenchyme. The formation of intermediate radials occurs through segmentation of an initially continuous cartilaginous precursor, a process reminiscent of joint development. Histological analysis of adult fins reveals that the *reph* intermediate radials form both proximal and distal epiphyses, and the articulation between the proximal and intermediate radials forms a distinct joint pocket between them, accompanied by specialized differentiation of the mesenchyme shaping the interface. In addition, muscle attachment sites were identified on the intermediate radials - features that are not typically characteristic of radials in Teleostei.

At the genetic level, it has been shown that *hox11* genes, which are involved in zeugopod development in terrestrial limbs, are also critical for the formation of these intermediate radials. In the case of a triple knockout of *hoxa11a*, *hoxa11b*, and *hoxd11a*, the development of intermediate radials was not observed. At the same time, *reph* and *wan* mutants exhibited increased expression levels of *hoxa11b*, consistent with the proposed role of *hox11* genes in the development of intermediate radials as homologs of the zeugopod.

A triple knockout of *hox13* paralogs from the *A* and *D* clusters - which are normally expressed in distal regions of the tetrapod limb and inhibit the expression of the more proximal *hox11* genes - in *Danio* enhanced the observed phenotype and increased the number of developing intermediate radials. Although teleost fins lack the clear proximodistal segregation of *hox11* and *hox13* expression domains typical of tetrapod limbs, this effect indicates the presence of regulatory interactions between these genes similar to those observed in terrestrial appendages. Overall, these findings reveal that, despite the loss of the metapterygium, teleost fins retain the developmental potential for proximodistal segmentation characteristic of terrestrial limbs, including regulatory interactions between key genetic modules and the joint differentiation program (Hawkins et al., 2021).

In summary, key features of *Hox* genes expression in tetrapod limb development include: (1) segregation of *hoxa11* and *hoxa13* expression domains during late proximodistal limb bud differentiation and (2) a second, late phase of *Hoxd* cluster genes expression exhibiting reversed collinearity relative to the early phase. The foundations of the regulatory mechanisms underlying the second phase of *Hoxd* genes expression are already present in cartilaginous fishes and may have existed at early stages of limb evolution. The pattern of *Hox* genes expression in the fins of *Neoceratodus* (Dipnoi) exhibits features indicative of a transitional state toward terrestrial limbs. On one hand, *Neoceratodus* displays a late phase of *hoxd13* expression and a proximodistal distribution of *meis* and *hoxa11* expression gradients; on the other hand, the separation of *hoxa11* and *hoxa13* expression domains, characteristic of terrestrial limbs, is absent, distinguishing its fins from true limbs.

One of the fundamental differences between a fin and a limb is the presence of exoskeletal elements (ceratotrichia or lepidotrichia), which form the distal portion of the fin. Key regulators of this distal exoskeletal region of the fin blade in fishes are the *and* genes - *and1/2* and *and3* - which are involved in AER development and in the formation of distal fin skeletal elements, the actinotrichia, *and* are hypothesized to have played an essential role in the fin-to-limb transition (Zhang et al., 2010). Knockdown of *and* genes leads to the loss of actinotrichia and disrupts the migration of mesenchymal cells that form lepidotrichia. Since *and* are absent from tetrapod genomes, their loss likely accompanied the reduction of fin rays during the fin-to-limb transition (Zhang et al., 2010).

Along the evolutionary lineage from fishes to terrestrial vertebrates, both *and* are present in the coelacanth genome. Lungfishes retain only one ortholog, *and1/2*, whereas both and genes have been lost in tetrapods (Wang et al., 2021). Thus, a sequential reduction of *and* can be observed along the lobe-finned lineage from coelacanths to terrestrial vertebrates.

### 8.2 Anteroposterior axis

A key regulator of anteroposterior patterning of the limb bud is the signaling center known as the zone of polarizing activity (ZPA), located in the posterior-distal mesenchyme and acting as a source of the morphogen Sonic hedgehog (Shh) (Riddle et al., 1993; Sheeba et al., 2016a). Shh secreted from the ZPA antagonizes the transcription factor Gli3, maintaining the balance between its activator (Gli3A) and repressor (Gli3R) forms (Litingtung et al., 2002; Te Welscher et al., 2002). Expressed in the posterior region of the limb, Shh suppresses the formation of the repressor form Gli3R in that region, thereby contributing to the formation of a gradient in the Gli3A:Gli3R ratio, with the highest level of the repressor form in the anterior part of the limb bud.

In the case of *Shh* knockout, the repressor form of Gli3 is produced throughout the limb, resulting in severe developmental defects of the zeugopod (absence or fusion of bones) and the autopod (formation of a single digit) (Litingtung et al., 2002). At the same time, *Gli3* knockout and double knockout of *Gli3* and *Shh* produce similar phenotypes - polydactyly (Litingtung et al., 2002; Te Welscher et al., 2002). These findings indicate that the Shh signal itself is not strictly required for the formation of limb segments; however, the Shh - Gli3 regulatory circuit plays a key role in the specification of the autopod and digits.

At the cellular level, *Shh* knockout leads to apoptosis, whereas suppression of *Gli3* restores cell survival. The functional role of Shh consists in antagonizing the repressor activity of Gli3, which in turn leads to the activation of genes involved in the growth and patterning of the limb bud, including *Hoxd11*, *Hoxa11*, *Hoxd13*, *Gremlin*, and *Fgf4* (Te Welscher et al., 2002). Subsequent studies expanded the list of genes dependent on Shh activity and, based on spatial expression patterns, identified three groups of genes: 1) genes of the posterior-distal region involved in transcriptional regulation and cell proliferation; 2) genes of the central region of the bud associated with cell differentiation and adhesion; 3) genes of the posterior-proximal region associated with skeletal development (Lewandowski et al., 2015). It was shown that genes of the first group require prolonged Shh signaling to maintain proliferation in the distal growth zone of the limb bud, whereas genes of the proximal and central regions require Shh only for their initial activation.

The search for GLI-binding regions (GBRs) revealed that acetylation of the majority of these regions (94%) does not depend on the presence of Shh; however, Shh-responsive enhancers were also identified (Lex et al., 2020). Such Shh-sensitive enhancers are typically located far from promoters and cluster in the genome near known Shh target genes such as *Grem1* and *Ptch1*. It was further shown that the level of acetylation at 88% of Shh-sensitive enhancers was restored to normal or even elevated levels in Shh/Gli3 double knockouts without requiring additional activator activity. These observations support the model according to which the principal role of Shh signaling in the limb bud is the removal of the repressive influence of Gli3 (Lex et al., 2020).

Functional studies of the Shh-Gli3 regulatory circuit have largely been conducted in the context of tetrapod limbs. This raises the question of when this regulatory circuit emerged during evolution: whether it is unique to terrestrial limbs or inherited from earlier appendage structures. In addressing this question, it was shown that disruption of *gli3* expression in the Japanese medaka (*Oryzias latipes*, Teleostei) leads to an increase in the number of both endoskeletal radials and external bony rays in fins - effects reminiscent of polydactyly in mice (Letelier et al., 2021). Suppression of *shh* expression through deletion of its ZRS enhancer resulted in the absence of pectoral fins (Letelier et al., 2018), whereas in double *shh/gli3* mutants the fins were restored and the phenotype resembled that of *gli3* mutants (Letelier et al., 2021).

The correspondence between the results obtained in a teleost model and those previously described in mice indicates that the Shh-Gli3 regulatory circuit arose in evolution prior to the emergence of terrestrial limbs and, given the detection of these signals in dorsal fins, possibly even before the origin of paired appendages (Letelier et al., 2021). At the same time, the range of functions of Shh-Gli signaling observed in fishes appears to include only the regulation of genes controlling cell proliferation, but not the specification of the anteroposterior axis of the fin bud, which involves genes such as *hand2* and *hox12a*. This suggests that during the evolution of terrestrial limbs the functional repertoire of the Shh-Gli3 regulatory circuit was expanded.

Among the targets of the posterior Shh signaling involved in limb bud differentiation are genes of the *Hoxa* and *Hoxd* clusters (Woltering et al., 2020; Leite-Castro et al., 2016). It has been demonstrated that disruptions in the activity of genes located in the 5′-region of the *Hoxa* and *Hoxd* clusters lead to limb reduction in mice (Dolle et al., 1993; Zakany et al., 1997; Boulet and Capecchi, 2004; Sheth et al., 2012). Experimental overexpression of the *hoxd13a* gene in *Danio* results in expansion of endochondral tissue and reduction of the apical fin fold in the fin bud, consistent with major morphological trends described for the fin-to-limb transition (Shubin et al., 2009; Freitas et al., 2012).

#### 8.2.1 Progressive posteriorization

A hallmark feature of patterning dynamics during limb ontogeny is “progressive posteriorization” whereby the expression domains of initially posterior genes (such as *hand2*) expand more intensely than anterior genes (e.g., *alx4*) as the limb bud develops (Figure 5C). This asymmetry results in a rotation of the original dorsal pattern towards the anterior, eventually occupying the distal position at later stages. In early fin buds of both actinopterygians and sarcopterygians, expression pattern of *hand2* in posterior part and *alx4* in anterior part resemble those in the terrestrial limb; however, progressive posteriorization is absent in fins, and the anteroposterior axis remains stable during development (Woltering et al., 2020).

Similar findings arise from comparisons of the genetic architecture of the catshark (*Scyliorhinus canicula*) fin and the mouse limb (Onimaru et al., 2015). Given the absence of anterior basal elements (propterygium and mesopterygium) in terrestrial vertebrates, the authors suggest that fin-to-limb transition involved modifications of anteroposterior regulatory networks. Expression domains of mouse anterior gene orthologs (*alx4, pax9, hand1,* and *zic3*) in the shark are shifted considerably posteriorly, whereas mouse posterior genes (*hand2* and *tbx2*) extend more anteriorly relative to shark orthologs (Onimaru et al., 2015). This anteriorized (compared to mouse) gene architecture in the shark fin is further supported by spatial distributions of the regulatory Gli3-Shh circuit, critical for anteroposterior differentiation. Notably, heterochrony (delayed onset) of *shh* expression in shark fins relative to terrestrial limbs has been proposed as a factor contributing to limb origin (Sakamoto et al., 2009). Additionally, *gli3* expression in shark fins is shifted posteriorly compared to terrestrial limbs, and its repression in the posterior limb domain is regulated by an enhancer element (*element 1586* or *hs1586*) found in terrestrial vertebrate genomes. It was found that *element 1586* is conserved in tetrapods, coelacanth, and chondrichthyans, but not in gar (representative of Holostei, diverged from Teleostei before TS-WGD), medaka, and zebrafish (Onimaru et al., 2015; Ali et al., 2024). The *element 1586* from chondrichthyans (*S. canicula* and *C. milii*) was shown to be capable of driving reporter GFP expression in chick limb buds, confirming that its general activity is conserved from sharks to tetrapods (Onimaru et al., 2015).

These data indicate that shifts in the spatial-temporal activity of preexisting regulatory mechanisms could drive the fin-to-limb transition. This is also supported by the results of transcriptomic comparisons between bamboo shark fins and mouse limbs (Onimaru et al., 2021). It has been shown that many genes whose activity increases at later stages of mouse limb development, associated with digit formation and tissue differentiation, exhibit stable expression throughout development in sharks, or their expression declines at later stages. The most evolutionarily conserved stages between sharks and mice correspond to the middle stages of fin/limb bud development. This pattern aligns with the “developmental hourglass” model, according to which the middle (phylotypic) stages are highly conserved, presumably due to the large number of regulatory interactions active during this period of embryogenesis and organ formation (Kalinka et al., 2010; Irie and Kuratani, 2014; Onimaru et al., 2021). Changes were also observed in the activity of the Shh signaling pathway. Although the *Shh* gene itself is activated at roughly the same relative developmental time in both shark and mouse limbs, the peak expression of its target genes (*ptch1*, *gli1*) in sharks coincides with the peak of *shh* itself, followed by a marked decline. In mice, by contrast, the peak activity of the target genes is delayed and occurs at a later stage, after *Shh* levels have already begun to decrease. This shift effectively extends the signaling window, which may be linked to the formation of the complex structures of the autopod (Onimaru et al., 2021). These findings indicate that the fin-to-limb transition may have been driven less by the emergence of new genes and more by a temporal reorganization of pre-existing developmental mechanisms (heterochrony) and an increase in their regulatory complexity.

The observed progressive posteriorization of regulatory gene expression in the terrestrial limb is consistent with the postaxial dominance hypothesis, which proposes that most mesopodial elements and all digits arise from the postaxial branch of the limb bud (Shubin and Alberch, 1986).

### 8.3 Dorsoventral axis

In addition to the proximodistal and anteroposterior axes, the terrestrial limb exhibits a pronounced dorsoventral subdivision, with distinct dorsal and ventral domains differing in muscle arrangement (flexors/extensors), skeletal structure, skin, and its derivatives (nails, pads, hair). Proper polarity is critical for limb function (Haro et al., 2021; Castilla-Ibeas et al., 2024).

Dorso-ventral patterning begins under the influence of somitic mesoderm, which instructs dorsal differentiation of adjacent LPM and, through its mediation, the overlying ectoderm, reflected in *wnt7a* expression on the dorsal side (Chen and Johnson, 2002). From the dorsal ectoderm, Wnt7a induces the expression of the LIM-domain transcription factor *lmx1b* in dorsal mesenchyme, which is necessary and sufficient for dorsal limb bud identity (Castilla-Ibeas et al., 2024). On the ventral side, BMP signaling induces the expression of *engrailed-1* (*en1*), which suppresses *wnt7a* activity, thereby spatially restricting its influence to the dorsal segment of the ectoderm (Cygan et al., 1997; Loomis et al., 1998; Chen and Johnson, 1999; Sheeba et al., 2016b). Expression of *Lmx1b* in mice begins prior to the appearance of the limb bud and is strictly restricted to the dorsal half of the bud, later persisting in the dermis beneath the nail and in the proximal nail fold even in adulthood (Riddle et al., 1995; Dealy et al., 1993; Parr et al., 1993; Castilla-Ibeas et al., 2024). *Lmx1b* knockout results in a double-ventral phenotype at the zeugopod and autopod levels, in which dorsal skeletal structures such as the patella are absent, whereas ventral structures, such as ventral sesamoid bones, are duplicated. In *Lmx1b*-null double-ventral limbs, tendons and muscles in the dorsal compartment adopt a symmetrical configuration, mirroring their ventral counterparts, and there is a loss of dorsal epidermal derivatives, namely hair and nails, while ventral ectodermal derivatives, such as pads and eccrine glands, develop on the dorsal side (Mahmud et al., 2022; Chen et al., 1998).

Conversely, *Lmx1b* overexpression in chick and mouse leads to a double-dorsal phenotype, indicating that *Lmx1b* is both necessary and sufficient for establishing dorsal limb morphology (Castilla-Ibeas et al., 2024). At the same time, *Lmx1b* is a highly pleiotropic gene with roles not only in limb dorsoventral patterning but also in the development of the central nervous system (CNS) and kidneys (Chen et al., 1998; Haldin et al., 2008).

The development of dorsoventral polarity is considered a key adaptation for the transition from fins to weight-bearing limbs, as observed in *Tiktaalik* (Shubin et al., 2006). This raises the question of at which evolutionary stage this dorsoventral asymmetry first arose. Recent studies have shown that a trend toward increased asymmetry between dorsal and ventral hemitrichia of the fin was already established prior to the origin of digits in fossil tetrapodomorphs (Stewart et al., 2020). This is further supported by the patterning of antagonistic abductor and adductor muscles with specialized motor neuron innervation (Zdral et al., 2026).

The evolutionary conservation of the dorsoventral pattern in paired fins is corroborated by the expression of *Lmx1b* in the dorsal portion of pectoral fins of *Danio* and the little skate *Leucoraja erinacea* (Uemura et al., 2005; Jung et al., 2018; Dale et al., 2026). In the ventral ectoderm of pectoral fins of *Danio*, as well as in basal fish lineages such as *Polyodon spathula* (Chondrostei), *Hemiscyllium ocellatum*, and *Callorhinchus milii* (Chondrichthyes), the expression of *wnt7aa* and *en1a* in dorsal and ventral ectoderm, respectively, has been reported, confirming the conservation of dorsoventral regulation in paired fins of fishes and terrestrial limbs (Dale et al., 2026).

A key question is whether the mechanism of dorsoventral differentiation is unique to paired fins or whether similar regulatory circuits arose earlier, in unpaired fins. In addressing this, expression of *lmx1b* orthologs has been described in the posterior mesenchyme of median (dorsal and anal) fins, corresponding to the ZPA, across phylogenetically diverse fish groups: *Danio* and *Tilapia* (Teleostei), *Acipenser baeri* and *P*. *spathula* (Chondrostei), and *Scyliorhinus canicula*, *H*. *ocellatum*, *C*. *milii* (Chondrichthyes) (Zdral et al., 2026; Dale et al., 2026; Höch et al., 2021). Expression in basal gnathostomes indicates the deep evolutionary origin of *lmx1b* regulatory activity in vertebrates. This is further supported by the expression of *lmx1b* orthologs in the posterior regions of the second dorsal and ventral fins in larvae of the sea lamprey (*Petromyzon marinus*), a modern jawless vertebrate and sister group to gnathostomes (Hawkins et al., 2025; Zdral et al., 2026).

Ectodermal expression of *wnt7a* has also been detected in dorsal and ventral fins of *Danio*, *P. spathula*, *H. ocellatum*, and *C. milii*, suggesting the possibility of regulatory interactions between spatially adjacent *lmx1bb* and *wnt7aa*, similar to those observed in the limb bud, even in unpaired fins (Dale et al., 2026). In contrast to paired appendages, no *en1* expression was detected in median fins, which in paired fins contributes to ventral specification (Dale et al., 2026). Cell lineage tracing of *lmx1bb*-expressing cells has shown that in both pectoral and dorsal fins, these cells contribute to the formation of endoskeletal radials and dermal hemitrichia (Dale et al., 2026).

## 9 Evolution of regulatory regions: conserved enhancers and novelties

### 9.1 Proximodistal axis–changes in *Hox* genes regulation

Regulatory mechanisms underlying the early and late phases of *Hoxd* expression involve two enhancers contained within the 3′regulatory domain (3DOM), responsible for proximal elements, are conserved between fishes and tetrapods, while the 5′domain (5DOM) had experienced a gain of regulatory regions linked to the evolution of the autopod (Woltering et al., 2014; Montavon et al., 2011; Andrey et al., 2013). The opposite positioning of these enhancers can explain differences in collinearity patterns observed during early versus late expression stages (Onimaru et al., 2021). Similar regulatory mechanisms have been described for the *Hoxa* cluster (Berlivet et al., 2013), and homologous regulatory elements have been identified in both gars (*Lepisosteus*) and teleosts (*Danio*) (Schneider et al., 2011; Woltering et al., 2014; Gehrke et al., 2015).

Deletion of the 3′DOM region in *Danio* resulted in a complete loss of expression of *hoxd4a* and *hoxd10a* in the fins, corresponding to the effects observed in mice and indicating the evolutionary conservation of regulatory mechanisms controlling the development of proximal limb and fin domains (Hintermann et al., 2025). In contrast, deletion of the 5′DOM in *Danio* had only a minor effect on *hoxd13a* expression in fins, unlike in mice, where 5′DOM deletion completely abolishes *Hoxd13* expression in digits (Hintermann et al., 2025).

Interestingly, 5′DOM deletion in fishes led to a complete loss of *hoxa13a*, *hoxa13b*, and *hoxd13a* expression in the developing cloacal region and caused defects in intestinal and renal duct development. This suggests that the 5′DOM elements may have originally regulated *hox13* genes in the cloaca, and during limb evolution, this regulatory module was co-opted to control the development of distal limb structures (digits) (Hintermann et al., 2025).

The absence of distal *hoxa11* expression in the tetrapod limb is attributed to an enhancer located within the *hoxa11* intron that activates antisense RNA transcription at the *hoxa11* locus under the influence of HOXA13 and HOXD13 proteins. This enhancer is absent in teleosts, where *hoxa11* and *hoxa13* expression domains overlap in fins (Kherdjemil et al., 2016).

The observed spatiotemporal expression patterns of *hoxa13* and *hoxd13* underscore their critical role in distal limb development (Zakany and Duboule, 1999). *Hox* clusters in *Danio* and mouse exhibit similar organization, reflecting high conservation and suggesting shared regulatory mechanisms. However, when expressed in transgenic mouse limbs, *Danio hoxa* and *hoxd* genes are active only proximally; expression in the autopod domain typical of mouse *hoxa13/hoxd13* is absent in *Danio* orthologs, indicating distinct regulatory control of *Hox* gene expression in distal tetrapod limbs (Woltering et al., 2014).

Recent genomic sequencing of lobe-finned fishes has drawn considerable attention as a means to identify genetic features reflecting the transitional status of their fins on the path to terrestrial limbs. Of six described enhancers regulating the late phase of *Hoxd* cluster expression, three are tetrapod-specific. One cis-regulatory element, *island1*, is shared between the coelacanth and terrestrial vertebrates but absent in ray-finned fishes (Amemiya et al., 2013). In mouse embryo experiments, the coelacanth *island1* sequence was able to specifically induce reporter gene expression in the limb bud, indicating its emergence at the ancestral lobe-finned stage. This supports the hypothesis that the autopod evolved through modulation of evolutionarily ancient regulatory mechanisms.

*Neoceratodus* fins exhibit expression of *Hoxc* genes previously described only in mammals, further indicating the genetic groundwork for limb emergence in ancestral groups. The genome of the African lungfish *Protopterus annectens* contains a conserved non-coding element (CNE), 67 nucleotides in length, similar to the enhancer described for *hoxa11* in terrestrial vertebrates (Wang et al., 2021).

### 9.2 Anterio-posterior axis - Shh/Gli3 regulation

In studies of the regulatory mechanisms controlling Shh expression, an enhancer element known as ZRS (ZPA Regulatory Sequence) was identified. This is a long-range enhancer that controls tissue-specific *Shh* expression and is located approximately 1 Mb from the *Shh* transcription start site, within the fifth intron of the *Lmbr1* gene (Lettice et al., 2003). Orthologous sequences of ZRS have been found in the genomes of both terrestrial vertebrates and bony and cartilaginous fishes (Lettice et al., 2003; Letelier et al., 2018; Ball et al., 2025). The *in vivo* activity pattern of this enhancer is conserved across a wide range of vertebrates, including fish, but not in snakes, where ZRS sequences carry snake-specific changes (Kvon et al., 2016). Deletion of ZRS in the medaka *Oryzias latipes* led to a moderate reduction of shh expression at 3 days post-fertilization (dpf) and mild phenotypic defects in pectoral fin development, in contrast to the effects observed in mice, where ZRS deletion results in complete limb loss (Letelier et al., 2018). Interestingly, this ZRS deletion caused an almost complete loss of the dorsal fin. It was further shown that shh expression in paired fins is additionally regulated by shadow enhancers (sZRS). These results indicate a deep evolutionary conservation of the basic mechanisms controlling the development of unpaired and paired fins, with the emergence of additional regulatory elements coinciding with the origin of paired appendages (Letelier et al., 2018).

In *Neoceratodus shh* expression is conserved in the ZPA region and is regulated also by ZRS located in the intron of the *LMBR1* gene (Schartl et al., 2024). In South American and African lungfishes the fins are reduced to filamentous outgrowths that lack radials. This reduction is associated with sequence changes in regulatory elements, including two enhancers of the *hoxa* gene cluster (e10 and mm406), and ZRS. Experiments with transgenic mice have confirmed the inability of the reduced ZRS regulatory elements from the South American lungfish to drive the reporter gene expression in the limb region in contrast to the Australian lungfish ZRS element. Treatment of regenerating fins in the African lungfish *Protopterus* with a Shh pathway agonist (SAG) led to partial restoration of lost skeletal elements, confirming the key role of *Shh* pathway modulation in the morphological transformation of fins in this species (Schartl et al., 2024).

The loss in South American lungfish of genes such as *PRKG2*, *RASGEF1B*, *TTC23*, and *hoxd12* may also be potentially related to the secondary reduction of lepidosirenid fins (Schartl et al., 2024).

One of Shh target genes, *sall1*, is expressed in the fins of the lungfish *Neoceratodus* and in the autopod of mice but is absent in the fins of teleosts (*Danio*) (Meyer et al., 2021). In mice, *Sall1* participates in limb bud development by modulating *Hox* genes expression (Kawakami et al., 2009). Expression of *sall1* in *Neoceratodus* fins, regulated by enhancer *hs72*, suggests the emergence of its regulatory role already in ancestral lobe-finned fishes (Meyer et al., 2021).

Enhancers (CNEs) contribute to the regulation of *GLI2* and *GLI3* gene activity in limb buds, some of which have been identified in cartilaginous fishes, indicating their deep evolutionary origin (Ali et al., 2021; Anwar et al., 2015).

The maintenance of the ZPA during limb development depends on a feedback loop in which *Shh* activates the secreted BMP inhibitor *Gremlin1*, which is required for *Fgf* expression in the AER (Scherz et al., 2004; Niswander et al., 1994). Studies of *Gremlin1* regulation within the *Shh-Grem1-Fgf* signaling loop, essential for growth and digit formation, revealed a network of multiple enhancers that control both the quantitative level of *Gremlin1* expression and its spatial patterning. Notably, disruptions of *Gremlin1*’s spatial activity have a stronger effect on autopod development than quantitative changes in expression levels (Malkmus et al., 2021). Two of these enhancers (CRM2 and CRM5) are present not only in all tetrapods but also in the coelacanth and sharks. Remarkably, the CRM2 enhancer from elephant and bamboo sharks, as well as coelacanth CRM2, retained functional activity (capable of driving reporter gene expression) in the mouse autopod, demonstrating the evolutionary conservation of this regulatory module (Malkmus et al., 2021).

In *Neoceratodus* fins, components of the *Shh-Gli3* axis and the *Shh-Grem1-Fgf8* feedback loop are expressed in a pattern consistent with a role in anterior-posterior patterning similar to that in tetrapod limbs, although possibly with distinct dynamics that could account for the different posteriorization of lungfish fins (Woltering et al., 2020).

### 9.3 Dorso-ventral axis - changes in *Lmx1b* regulation

Spatial expression data of key dorsoventral limb regulators - *lmx1b*, *wnt7a*, and *en1* - in basal gnathostomes indicate that the regulatory interactions among these factors were already present in paired fins and may have formed long before the emergence of terrestrial limbs (Zdral et al., 2026; Dale et al., 2026). The precursors of these regulatory circuits may have arisen even prior to paired fins, in median fins (Zdral et al., 2026; Dale et al., 2026). At the same time, the regulatory context of these factors could have been extensively remodeled during evolution as new morphological structures appeared.

Expression of *Lmx1b* in paired appendages is regulated by Wnt signals (*Wnt7a*, *Wnt7b*) via the β-catenin pathway (Adamska et al., 2004; Adamska et al., 2005). Additionally, two autoregulatory enhancers (*LARM1* and *LARM2*) maintain its own expression (Haro et al., 2021). Double-ventral limbs in Δ*LARM1/2* mutants fail to support locomotion and are even incapable of lifting body weight (Haro et al., 2021).

In unpaired fins, *lmx1b* expression is regulated by *shh*, rather than *wnt7a* or *en1* (in contrast to paired fins) (Zdral et al., 2026). Suppression of Wnt signaling did not affect *lmx1bb* expression in unpaired fins (this paralog arose in Teleostei after the teleost-specific WGD), whereas *shh* inhibition significantly reduced *lmx1b* levels in the ZPA region of dorsal and anal fins (Zdral et al., 2026). Activation of *shh* led only to a modest increase in *lmx1bb* expression, indicating that *shh* is just one component of the activator module controlling *lmx1b* expression.

Enhancers *LARM1* and *LARM2*, which regulate *Lmx1b* expression, were first described in mice, where their activity is specific to the regulation of *Lmx1b* in limbs (Haro et al., 2021). Deletion of *LARM1/2* results in double-ventral limbs, while other structures such as kidneys, eyes, and brain develop normally (Haro et al., 2021). Spatial subfunctionalization along the anteroposterior axis was observed between the two enhancers: *LARM1* contributes primarily to dorsalization of the posterior limb, whereas *LARM2* affects the anterior limb (Haro et al., 2021). The functional role of these *LARM* enhancers was further supported by analysis of patients with Nail-Patella Syndrome (NPS), an inherited disorder characterized by hypoplastic nails and patellae, in which a *LARM2* deletion was identified (Haro et al., 2021).

Sequences similar to *LARM1* have been found in representatives of ray-finned and cartilaginous fishes, and *LARM2* in ray-finned fishes (Hawkins et al., 2025; Dale et al., 2026). These *LARM* elements were capable of driving spatially specific expression of fluorescent reporter proteins in paired fins of *Danio* (Hawkins et al., 2025). Deletion of *LARM1* and *LARM2* sequences in *Danio* led to reduced *lmx1bb* expression in paired fins and the development of a double-ventral phenotype, with *LARM1* deletion contributing the most (Dale et al., 2026; Hawkins et al., 2025). In these mutants, *lmx1bb* expression in unpaired fins and their normal morphology were maintained (Dale et al., 2026; Hawkins et al., 2025).

In addition, in the shark *H*. *ocellatum*, *LARM*-dependent regulation of *lmx1b* was shown to participate in dorsal fin development within a more complex regulatory network than in paired fins (Dale et al., 2026). These findings indicate a broad evolutionary conservation of the contribution of *LARM* enhancers to *lmx1b* regulation in gnathostomes, prior to the emergence of terrestrial-type limbs.

The similarity of genetic modules controlling proximodistal, anteroposterior, and, to some extent, dorsoventral axes in paired and unpaired fins supports the hypothesis that paired fins originated via co-option of regulatory mechanisms from evolutionarily older unpaired fins (Woltering and Duboule, 2010; Woltering et al., 2020; Zdral et al., 2026). At the same time, by the stage of paired fin emergence, pre-existing regulatory mechanisms had already undergone modifications, particularly involving the establishment and enhancement of dorsoventral asymmetry, which was fully realized during the fin-to-limb transformation.

## 10 Did the tetrapod autopodium arise *de novo*, or does it have deep evolutionary roots?

As we noted above, the most debated question in the context of the fin-to-limb transition concerns the origin of the distal segment, the autopodium, structurally comprising the manus/pes elements and digits.

Classical hypotheses (e.g., Westoll, 1943; Shubin and Alberch, 1986) propose that digits derive from distal radials of a biserial fin. One hypothesis posits that the autopodium may have formed through the involvement of a regulatory exoskeletal mechanism that was modified following the reduction of fin rays (Nakamura et al., 2016). Supporting this is the expression of the *hoxa13* gene observed in the fin fold of both actinopterygian and sarcopterygian fishes, as well as in the autopodium of the terrestrial limb (Woltering et al., 2020). Experimental knockdown of *hoxa13* in *Danio* led to a reduction of the exoskeletal fin fold and an increase in the number of distal endoskeletal radials, consistent with major morphological trends in the fin-to-limb transition and suggestive of conserved regulatory foundations between these two limb types (Nakamura et al., 2016). An alternative scenario suggests that digits arose through the evolution of novel regulatory mechanisms that established the characteristic two-phase expression of *Hoxd* genes in the limb autopodium (Woltering et al., 2014).

Computed tomography analysis of the pectoral fin of the fossil *Panderichthys* revealed the complexity of its distal fin skeleton. A cluster of small bones was identified, including a small ulnare and several distal radials comparable to tetrapod digits (Boisvert et al., 2008). The fin skeletons of two other elpistostegalians, *Tiktaalik* and especially *Elpistostege*, were also found to be highly segmented (Shubin et al., 2006; Cloutier et al., 2020). *Tiktaalik*’s fin not only bore a series of radials but also possessed synovial joints similar to those in tetrapod limbs, allowing it to support itself on the substrate by flexing the elbow joint and spreading the distal fin over the surface (Shubin et al., 2006). Recent studies have shown that synovial joints are not unique to terrestrial limbs but appeared in evolution well before the colonization of land, representing a gnathostome synapomorphy, supported by both molecular and paleontological evidence (Sharma et al., 2025; Askary et al., 2016).

In *Elpistostege*, the distal fin included four transverse rows of radials; the two proximal rows likely correspond to mesopodial elements, while the two distal rows correspond to digital phalanges (Cloutier et al., 2020). In the fins of modern *Neoceratodus*, distal radials are present on both the preaxial and postaxial sides of the central axis. Despite structural and developmental similarities of such fins with terrestrial limbs, their ontogeny lacks the progressive posteriorization characteristic of the tetrapod autopod (Woltering et al., 2020).

Taken together, data from the fins of the closest fish relatives of tetrapods, combined with developmental data from basal actinopterygians, sharks, and lungfish, suggest that digits may not be a tetrapod novelty but could have originated from pre-existing distal fin radials of lobe-finned fishes (Davis et al., 2007; Dahn et al., 2007; Johanson et al., 2007; Boisvert et al., 2008; Cloutier et al., 2020). Regulatory modules involved in digit development in terrestrial limbs may have evolved long before these morphological structures appeared and were co-opted for their formation at a certain evolutionary stage (Hintermann et al., 2025).

At the same time, mesopodial elements of the middle and distal rows, unlike digits, have in recent decades often been recognized as novel structures that evolved during tetrapod limb evolution (Johanson et al., 2007). Based on the limbs of Devonian tetrapods, developed digits appeared earlier than a fully formed mesopodium consisting of several centralia and a complete series of distal elements (Coates, 2003; Clack, 2004; 2012; Johanson et al., 2007). The emergence of a full set of distal and central mesopodial elements may have resulted from the addition of new centralia distal to the intermedium and radiale/tibiale, and a series of independent ossifications (distal elements) at the bases of digits (Johanson et al., 2007). In *Neoceratodus*, all fin radials except the first preaxial one, as well as salamander digits, initially form independently from other skeletal elements from a separate skeletogenic tissue (Holmgren, 1933). The independent formation of radials in *Neoceratodus* correlates well with the expression of the *hoxd13* gene and the late phase of *Hox* genes expression during tetrapod autopodium formation (the features of *Hox* genes expression in limb development will be discussed in detail below) (Johanson et al., 2007).

Descriptions of the *Elpistostege* fin skeleton demonstrated that many skeletal elements considered characteristic only of tetrapod limbs were already present in its fins. *Elpistostege* possessed a probable homolog of the carpals and two distal rows of mesopodial elements (comparable to tetrapod centralia and distal elements), in addition to proximal ulnare and intermedium (Cloutier et al., 2020). Distal to the mesopodium, the fin also bore digits, some composed of two phalanges. The sole principal distinction of *Elpistostege*’s fin from a typical tetrapod limb was the presence of lepidotrichia. These data support the interpretation of *Elpistostege* as the first true tetrapod, with its pectoral fins representing a transitional form from fins to primitive tetrapod limbs, requiring no additional assumptions about major novel skeletal elements in the autopodial region (Cloutier et al., 2020).

## 11 Self-organization in autopod development - the Alan Turing model

Morphogenesis is a dynamic process in which cells differentiate and organize into tissues that form three-dimensional structures and specialized organs. The high reproducibility of phenotypes indicates that development is strictly controlled in space and time, with cells producing and receiving specific information that organizes their behavior (Collinet and Lecuit, 2021). Already in the late 19th century, Hans Driesch and Thomas Morgan demonstrated that embryos of sea urchins and amphibians, when bisected, are capable of reprogramming their development to regenerate the missing part (De Robertis, 2006). This suggested that during development, cells can interact with one another and, upon receiving external cues, develop in directions that are not predetermined. Such a capacity for self-organization laid the foundation for the regulative view of development. Today, it is clear that morphogenesis relies on both deterministic programs and self-organization (Collinet and Lecuit, 2021). This duality may also be present in limb development. In this context, to explain the mechanism of digit formation in the autopodium, a hypothesis based on Alan Turing’s mathematical model of self-organizing interactions between two morphogens—an activator and an inhibitor with differing diffusion rates - was proposed (Turing, 1952; Newman and Frisch, 1979). The Turing model itself describes a possible mechanism for the formation of repeating patterns, and similar regulatory circuits have been reported not only in digit formation, but also in the establishment of skin patterns in vertebrates, feather follicle patterns in chick, and palatal and hair patterns in mice (Watanabe and Kondo, 2015; Jung et al., 1998; Economou et al., 2012; Sick et al., 2006; Kondo, 2022). Experimental validation of digit patterning based on the Turing model emerged from polydactyly phenotypes in mice with reduced *Hox* gene signaling combined with knockout of the *Shh-Gli3* regulatory axis (Sheth et al., 2012). Subsequently, factors such as **B**MP, **S**ox9 and **W**nt (**BSW**-model) were implicated as candidate morphogens involved in digit development (Raspopovic et al., 2014). Subsequently, in Chondrichthyes, *sox9* expression was described in the distal regions of developing endoskeletal fin elements, along with a regulatory circuit similar to the BSW observed in mice, suggesting the evolutionary antiquity of this regulatory mechanism in gnathostomes (Onimaru et al., 2016).

Paleontological data indicate that terrestrial limbs evolved with varying digit numbers, but the pentadactyl limb became the conserved ground plan (Laurin et al., 2000; Clack, 2009; Coates and Clack, 1990). In species with digit reduction (e.g., ungulates), early limb buds initially form five digits, with digit loss occurring during development (Kavanagh et al., 2020). Within the Turing framework, digit number depends on parameters such as morphogen diffusion rates, binding constants, and limb bud growth rates.

The BSW mechanism is also proposed to operate in the development of radials in the caudal fin of teleosts (Desvignes et al., 2022). Interestingly, in this study, the formation of an externally symmetric fin (at late stages) begins with the formation of hypurals on the ventral side of the notochord, followed by dorsal bending of the caudal end - a process visually reminiscent of the proposed curvature of the metapterygial axis (see Figure 2 in Desvignes et al., 2022).

While the real regulatory mechanisms underlying the development of complex morphological structures are multi-component and do not always fit neatly within theoretical models, we suggest that self-organization represents one productive conceptual avenue guiding experimental work. A coherent and non-contradictory synthesis of experimental data with evolutionary concepts developed through comparative morphology and paleontology constitutes, in our view, one of the major challenges in understanding the fin-to-limb transition in the near future.

## 12 Conclusion

The fin-to-limb transition represents one of the most consequential evolutionary transformations in vertebrate history, enabling the emergence of tetrapods and the colonization of terrestrial environments. Evidence from paleontology, comparative anatomy, and developmental genetics indicates that this transition occurred through a gradual modification of ancestral paired fins rather than through the sudden appearance of entirely novel structures. Fossil tetrapodomorphs such as *Panderichthys*, *Tiktaalik*, and *Elpistostege* demonstrate that several key tetrapod-like features - including elaborated endoskeletal elements and partial reduction of dermal fin rays and dorso-ventral specification arose in aquatic forms prior to the origin of fully digit-bearing limbs. At the same time, recent developmental and genomic studies show that the genetic regulatory networks controlling appendage formation, including AER (*Fgf*), ZPA (*Shh-Gli3*), and *Hox* signaling modules, are deeply conserved across gnathostomes and were progressively modified during the evolution of tetrapod limbs. Taken together, current evidence indicates that morphological and genetic foundations of tetrapod limbs originated in aquatic sarcopterygians prior to terrestrialization. The fin-to-limb transition resulted primarily from evolutionary modifications of conserved developmental regulatory networks rather than from the emergence of entirely novel genetic mechanisms. The most significant evolutionary innovations during the fin-to-limb transition occurred in the distal appendage region, where dermal fin rays were reduced and the endochondral skeleton expanded to form the autopod and digits.

Studies of phylogenetically pivotal extant groups provide additional insight into the evolutionary steps underlying this transformation. Cartilaginous fishes retain ancestral features of appendage organization and regulatory architecture, whereas lungfishes exhibit developmental and morphological characteristics that approach the tetrapod condition. In particular, the biserial organization of lungfish fins and their regulatory gene expression patterns suggest that elements of the tetrapod limb developmental program were already present in the last common ancestor of sarcopterygians.

Despite significant advances in recent years, several key questions remain unresolved. In particular, the precise homology of distal skeletal elements between fins and limbs, the developmental mechanisms underlying autopod emergence, and the evolutionary modifications of regulatory networks that generated digit patterning continue to be actively debated. Further integration of paleontological discoveries with comparative genomics and functional developmental studies in phylogenetically pivotal taxa will continue to refine our understanding of how vertebrate fins were transformed into the limbs that ultimately enabled the radiation of terrestrial vertebrates.
